# Inflammation and Oral Contraceptive Use in Female Athletes Before the Rio Olympic Games

**DOI:** 10.3389/fphys.2020.00497

**Published:** 2020-05-25

**Authors:** Brianna Larsen, Amanda Cox, Candice Colbey, Michael Drew, Helen McGuire, Barbara Fazekas de St Groth, David Hughes, Nicole Vlahovich, Gordon Waddington, Louise Burke, Bronwen Lundy, Nicholas West, Clare Minahan

**Affiliations:** ^1^Griffith Sports Physiology and Performance, School of Allied Health Sciences, Griffith University, Gold Coast, QLD, Australia; ^2^Menzies Health Institute Queensland, Griffith University, Gold Coast, QLD, Australia; ^3^Queensland Academy of Sport, Nathan, QLD, Australia; ^4^School of Medical Sciences, Griffith University, Gold Coast, QLD, Australia; ^5^Australian Institute of Sport, Canberra, ACT, Australia; ^6^Australian Centre for Research into Injury in Sport and its Prevention (ACRISP), Federation University Australia, Ballarat, VIC, Australia; ^7^University of Canberra Research Institute for Sport and Exercise, Canberra, ACT, Australia; ^8^University of Sydney, Sydney, NSW, Australia; ^9^Centenary Institute, Sydney, NSW, Australia

**Keywords:** C-reactive protein, cytokines, contraception, athletes, sex hormones

## Abstract

This study investigated the association between synthetic ovarian hormone use [i.e., the oral contraceptive (OC) pill] and basal C-reactive protein (CRP), peripheral blood immune cell subsets, and circulating pro- and anti-inflammatory cytokine concentrations in elite female athletes. Elite female athletes (*n* = 53) selected in Rio Summer Olympic squads participated in this study; 25 were taking an OC (AthletesOC) and 28 were naturally hormonally cycling (AthletesNC). Venous blood samples were collected at rest for the determination of sex hormones, cortisol, CRP, peripheral blood mononuclear memory and naïve CD4+ T-cells, CD8+ T-cells and natural killer cells, as well as pro- and anti-inflammatory cytokine concentrations. C-reactive protein concentrations were elevated (*p* < 0.001) in AthletesOC (median = 2.02, IQR = 3.15) compared to AthletesNC (median = 0.57, IQR = 1.07). No differences were reported for cortisol, cytokines, or PBMC immune cell subsets, although there was a trend (*p* = 0.062) for higher IL-6 concentrations in AthletesNC. Female Olympians had substantially higher CRP concentrations, a marker of inflammation and tissue damage, before the Rio Olympic Games if they used an OC. Future research should examine the potential consequences for athlete performance/recovery so that, if necessary, practitioners can implement prevention programs.

## Introduction

Athletes are more susceptible to illness and infection during periods of intense training and competition (Walsh and Whitham, 2006; Gleeson, 2007), which may be compounded in an Olympic environment. Data from the London 2012 Olympic Games found that female athletes were 60% more likely to fall ill than male athletes, and one in five illnesses was expected to result in absence from training/competition (Engebretsen et al., 2013). This absence is associated with decreased performance outcomes (Drew et al., 2017a). Thus, factors that contribute to an increased risk of infection/illness in elite female athletes must be further examined so practitioners can implement prevention programs.

Female sex hormones play an integral role in many physiological pathways, including regulation of the immune system (Schuurs and Verheul, 1990). There is evidence to suggest that the majority of female athletes use some form of oral contraceptive (OC) (Rechichi and Dawson, 2009), which reduces the concentration and cyclical variability of endogenous estrogen and progesterone (Fleischman et al., 2010). While the OC pill may be beneficial for sportswomen by reducing menstrual cycle variability, it is possible that the administration of synthetic sex steroids interferes with normal immune homeostasis.

C-reactive protein (CRP) is an acute phase reactant used as a systemic marker of inflammation and tissue damage (Pepys and Hirschfield, 2003). Elevated CRP concentrations are also associated with an increased risk of developing cardiovascular disease (CVD) (Ridker et al., 2000) and diabetes mellitus (Freeman et al., 2002). When assessing CVD risk in healthy women, CRP thresholds of <0.5 mg.L^–1^ (protective), 0.5–1.0 mg.L^–1^ (no risk), 1.0–3.0 mg.L^–1^ (intermediate risk), 3.0–10.0 mg.L^–1^ (high risk), and >10.0 mg.L^–1^ (very high risk) have been utilized (Cook et al., 2006; Cauci et al., 2017). Of interest, OC use has been associated with increased CRP concentrations in healthy female adolescents (Pirkola et al., 2010) and women (van Rooijen et al., 2006; Cauci et al., 2008; Piltonen et al., 2012; Sørensen et al., 2014; Cauci et al., 2017). However, only one of these studies (Cauci et al., 2017) utilized an athletic population, of which only 14.6% had competed at a national/international level (*n* = 30). Regular OC use has also been associated with a higher CD8+ T-cell number and lower natural killer cell number (Auerbach et al., 2002), suggesting that it may modulate basal immune status.

Regular physical activity promotes an anti-inflammatory immune profile (Petersen and Pedersen, 2005; Gleeson, 2007), and so it is possible that the effects of OC use on inflammation differ in elite sportswomen when compared to the general community. While evidence suggests that OC use is similarly associated with elevated CRP concentrations in athletic women (Cauci et al., 2017), further studies need to be performed utilizing elite-level female athletes to confirm and extend upon these findings. Therefore, this study measured basal CRP and peripheral blood mononuclear cells (PBMCs), as well as other markers of stress and inflammation (e.g., cortisol, pro- and anti-inflammatory cytokines), to provide a snapshot of the athletes’ inflammatory status prior to the 2016 Summer Olympic Games in Rio.

## Materials and Methods

Elite female athletes (*n* **=** 53) preparing for the Rio 2016 Olympics participated in this study [representing a subset of the Stay Healthy project; Phase 1 of the project is described elsewhere (Drew et al., 2017a, b)]. Athlete demographic information is presented in Table 1. Twenty-five athletes were taking OC (AthletesOC) and 28 were naturally cycling (AthletesNC); the type(s) of OC used by AthletesOC were not specified. AthletesOC has been taking OC for 6.5 ± 3.9 y, although it should be noted that only 12 of the 25 OC users provided information regarding length of OC use. Nonsteroidal anti-inflammatory use was reported by only three athletes (AthletesNC = 1, AthletesOC = 2). Participants completed the valid and reliable Low Energy Availability in Females Questionnaire (LEAF-Q) (Melin et al., 2014) and an illness questionnaire (to confirm they were healthy), and provided a single blood sample. Ethical approval was granted by the Australian Institute of Sport and the Griffith University Human Ethics Committee. All athletes provided written informed consent.

**TABLE 1:** Demographic information for AthletesOC and AthletesNC.

	AthletesOC	AthletesNC
Age (y)	24.7 ± 3.5	24.4 ± 4.0
Height (cm)	170.88 ± 7.64	171.78 ± 6.45
Weight (kg)	66.77 ± 7.31	69.13 ± 8.71
BMI	19.51 ± 1.60	20.08 ± 2.11
Sport		
Hockey	9	2
Rowing	1	3
Soccer	5	12
Water polo	5	4
Rugby 7’s	5	5
Triathlon	0	2

Blood samples were collected from the antecubital vein using standard venipuncture techniques. Sample times ranged from 8:00 a.m. to 3:00 p.m. but were consistent across each sport (i.e., all water polo players had samples taken at ∼11:00 a.m.), resulting in a similar spread of sample times across AthletesOC and AthletesNC. Some athletes did not train prior to sample collection, while others performed light training or a regular training session. However, the number of athletes in each category was similar across groups; 50, 29, and 21% of AthletesNC performed no training, light training, and regular training, respectively, compared to 56, 24, and 20% of AthletesOC. On average, AthletesNC had their blood taken 2.5 h post-training, which was very similar to AthletesOC (2.0 h). Blood samples were taken randomly with respect to menstrual cycle, however, hormone concentration data (progesterone and estradiol) were used to stratify AthletesNC according to menstrual cycle phase using established reference values (Stricker et al., 2006; data were converted to relevant units for comparison where necessary).

Serum concentrations of estradiol, progesterone, and free testosterone were determined using commercially available enzyme-linked immunosorbent assay (ELISA) kits (Abcam, Cambridge, United Kingdom) according to the manufacturer’s instructions. Serum cortisol concentrations were also determined using a commercially available ELISA kit (Abnova, Taipei City, Taiwan) according to manufacturer’s instructions. Inter-assay coefficients of variation were 9.7% for estradiol, 6.0% for progesterone, 8.1% for free testosterone, and 5.1% for cortisol. All samples were analyzed in duplicate.

Serum CRP concentrations were determined via an immunoturbidimetric assay using commercially available reagents and a COBAS Integra 400 system (Roche Diagnostics, Mannheim, Germany). Serum cytokine concentrations were assessed using commercially available 27-plex suspension array kits (Bio-Rad Laboratories Pty Ltd.; Hercules, CA, United States). This panel includes key pro- (IL1β, IL6, IL-8 TNF-α) and anti-inflammatory cytokines (IL-1ra, IL-10) as well as relevant growth factors (VEGF, PDGF) and regulatory cytokines (IFN-γ, IL-17). Assays were completed using a Bioplex 200 Suspension Array Reader (Bio-Rad Laboratories Pty Ltd.) according to the manufacturer’s instructions. Standard curves of cytokine concentration vs. fluorescence intensity were automatically generated by the Bioplex Manager Software (Bio-Rad Laboratories Pty Ltd.), and sample concentrations for each cytokine were extrapolated from respective standard curves. All samples were analyzed in duplicate and allocation of samples from AthletesOC and AthletesNC groups were counterbalanced between plates. The mean inter-assay coefficient of variation for analytes included in statistical analysis was 12.7% (range: 9.9–18.1%). Those analytes where a high proportion of samples with concentrations below limits of detection, or where calculated concentrations for a given analyte were based largely on extrapolation beyond the range of the standard curve, were not included in statistical analysis.

PBMCs were isolated as previously published (West et al., 2016). Briefly, PBMCs were isolated by Ficoll (GE Healthcare, United Kingdom) density gradient separation, washed twice with PBS at 4°C, suspended at a concentration of 1–2 × 106 cells/mL in medium containing 10% DMSO, and cooled to −80°C at a rate of −1°C per min before transfer to liquid nitrogen for storage until assay. Cryopreserved PBMCs were thawed and washed in with RPMI1640 (Thermo Fisher Scientific, Waltham, MA, United States) with 10% heat-inactivated fetal bovine serum. PBMCs were stained for mass cytometry analyses as described (Newell et al., 2012) with the antibodies listed in Table 2. Data were acquired on a CyTOF 2 Helios upgraded instrument (Fluidigm, Toronto, Canada) at the Ramaciotti Facility for Human Systems Biology, Sydney, Australia. Flow Cytometry Standard (FCS) files were analyzed with FlowJo X 10.0.7r2 (FlowJo, LLC, Ashland, OR, United States).

**TABLE 2:** Mass cytometry antibody panel.

Isotope	Antibody	Antibody source	Receptor
89Y	CD45	Fluidigm	Cell marker
143Nd	CD45RA	Fluidigm	CD4^+^ and CD8^+^ memory/naïve maker
145Nd	CD4	Fluidigm	CD4^+^ T-cell marker
148Nd	CD16	Fluidigm	NK-cell marker
149Sm	CD56	Fluidigm	NK-cell marker
170Er	CD3	Fluidigm	T-cell marker
168Er	CD8	Fluidigm	CD8^+^ T-cell marker

Statistical analyses were performed using the Statistical Package for the Social Sciences (SPSS V.24.0, Champaign, IL, United States) and Prism v7.00 (GraphPad Software Inc., San Diego, CA, United States). The distribution of the data was evaluated using Shapiro-Wilk tests. All hormone and cytokine markers (aside from RANTES) were not normally distributed, thus these data were analyzed using Mann Whitney U tests. These variables were reported as median values (interquartile range; IQR), where IQR equals the difference between the 75th and 25th quartiles. Mass cytometry data was normally distributed and, along with participant demographics (i.e., age, height, weight, BMI) and RANTES, were assessed using independent samples *t*-tests; these data were reported as mean ± standard deviation. Significance was set at *P* < 0.05.

## Results

There was no difference (*p* > 0.05) in age, height, weight, or BMI between AthletesNC and AthletesOC (Table 1). AthletesNC had higher concentrations of estradiol (*p* < 0.001), testosterone (*p* = 0.004), and progesterone (*p* = 0.001), but not cortisol (*p* = 0.41), when compared to AthletesOC (Table 3).

**TABLE 3:** Hormone concentrations for AthletesOC and AthletesNC.

	AthletesOC	AthletesNC
Estradiol (pg/mL)	6.12 (23.92)	35.01 (40.57)
Free testosterone (pg/mL)	0.41 (0.40)	0.80 (0.88)
Progesterone (ng/mL)	0.28 (0.46)	1.14 (12.07)
Cortisol (μg/dL)	12.23 (6.85)	10.40 (6.94)

*Data are presented as median (IQR).*

Estradiol and progesterone concentrations showed that in the AthletesNC group, 14 athletes had hormone concentrations commensurate with the follicular phase [estradiol: 155.00 (240.00) pmol/L, progesterone: 1.52 (4.66) nmol/L], 12 athletes had hormone concentrations commensurate with the luteal phase [estradiol: 225.00 (225.00) pmol/L, progesterone: 74.05 (58.33) nmol/L], and two athletes had hormone concentrations commensurate with ovulation [estradiol: 1120.00 (460.00) pmol/L, progesterone: 3.07 (1.46) nmol/L]. There were no differences between AthletesNC in the follicular and luteal phases of the menstrual cycle for cortisol (*p* = 0.471), CRP (*p* = 0.643), or any other cytokine (*p* > 0.05), with the exception of IL-6 (*p* = 0.009) and RANTES (*p* = 0.022). IL-6 was higher in the luteal phase [4.90 (2.17) pg/mL] when compared to the follicular phase [1.47 (2.57) pg/mL], whereas RANTES was elevated in the follicular phase (10216.30 ± 2383.60 pg/mL) when compared to the luteal phase (8156.43 ± 1786.71 pg/mL).

There were no differences (*p* > 0.05) between AthletesOC and AthletesNC for any of the cytokines detected by the assay, although there was a trend (*p* = 0.062) for higher IL-6 concentrations in AthletesNC (Table 4). Certain cytokines (e.g., IL-2, IL-4, IL-5, IL-7, IL-9, IL-12, IL-13, IL-15) and growth factors (e.g., GM-CSF, MCP-1, VEGF) were below the limits of detection for the majority of samples and were therefore not included in the analysis.

**TABLE 4:** Comparison of cytokine concentrations between AthletesOC and AthletesNC.

	AthletesOC	AthletesNC	*p*-value
IL-1β (pg/mL)	0.91 (0.67)	1.02 (1.79)	0.229
IL-1ra (pg/mL)	30.90 (39.65)	29.06 (22.15)	0.310
IL-6 (pg/mL)	1.22 (1.60)	3.88 (3.61)	0.062
IL-8 (pg/mL)	7.11 (9.83)	10.43 (9.69)	0.662
IL-10 (pg/mL)	5.60 (3.35)	6.12 (4.20)	0.687
IL-17 (pg/mL)	154.03 (133.32)	146.59 (63.98)	0.894
TNFα (pg/mL)	26.63 (31.31)	28.40 (87.42)	0.247
IFNγ (pg/mL)	29.82 (16.84)	27.17 (16.12)	0.940
PDGF (pg/mL)	2143.33 (1464.11)	2111.94 (832.51)	0.460
RANTES (pg/mL)	9460 ± 2511.97	9259.31 ± 2261.03	0.760

*IL-1ra, interleukin-1 receptor agonist; TNFα, tumor necrosis factor alpha; IFNγ, interferon gamma; PDGF, platelet-derived growth factor; RANTES (CCL5), regulated on activation, normal T cell expressed and secreted. Data presented as median (IQR), except for RANTES which is presented as mean ± standard deviation.*

**FIGURE 1:**
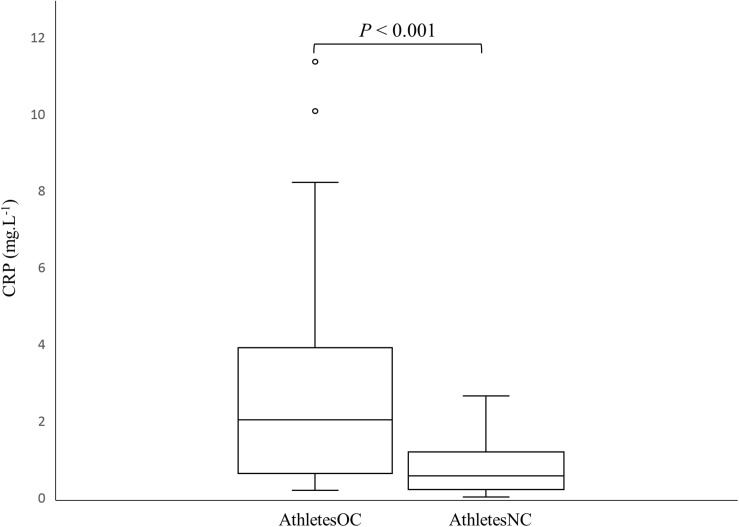
Box and whisker plot of C-reactive protein (CRP) concentrations in AthletesNC and AthletesOC. The *boxes* span the interquartile range (IQR), the *line* depicts the median value, the *whiskers* represent the highest and lowest values within a 1.5 IQR of the nearest quartile, and the *circles* depict the outliers.

Figure 1 shows the difference (*p* < 0.001) in CRP concentrations between AthletesOC and AthletesNC. The number of athletes in each CRP risk stratification category is also presented (Table 5).

**TABLE 5:** The proportion of AthletesOC and AthletesNC in each CRP risk stratification category.

Risk stratification	AthletesOC *n* (%)	AthletesNC *n* (%)
<0.5 mg.L^–1^ (protective)	4 (16)	11 (39)
0.5–1.0 mg.L^–1^ (no risk)	4 (16)	8 (29)
1.0–3.0 mg.L^–1^ (intermediate risk)	8 (32)	9 (32)
3.0–10.0 mg.L^–1^ (high risk)	7 (28)	0 (0)
>10.0 mg.L^–1^ (very high risk)	2 (8)	0 (0)
Total	25 (100)	28 (100)

**FIGURE 2:**
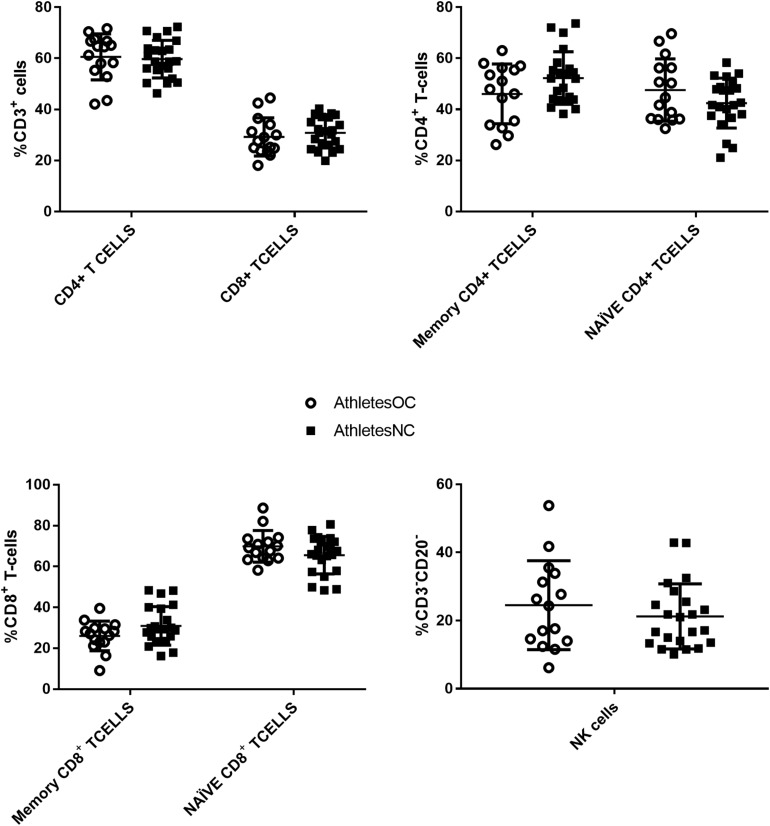
PBMC subsets between AthletesOC and AthletesNC. There were no significant differences between the groups in CD4^+^ or CD8^+^ T-cells **(top left)**, memory (CD3^+^, CD4^+^, CD45RO^+^) or naïve (CD4+, CD45RO^–^) CD4^+^ T-cells **(top right)**, memory (CD3^+,^ CD8^+^, CD45RO^+^) or naïve (CD3^+,^ CD8^+^, CD45RO^–^) CD8^+^ T-cells **(bottom left)**, or NK-cells (CD20-, CD56+, HLADR−) **(bottom right)**.

Immunophenotyping of PBMCs did not reveal differences between AthletesOC and AthletesNC. Figure 2 displays the frequency of CD4+ T-cells, memory (CD3+, CD4+, CD45RO+) and naïve CD4+ (CD3+, CD4+, CD45RO−) CD4+ T-cells, CD8+ (CD3+, CD8+) T-cells and memory (CD3+, CD8+, CD45RO+) and naïve (CD3+, CD8+, CD45RO−) CD8+ T-cells, and NK-cells (CD3-, CD20-, CD56+, HLADR−) for AthletesOC compared to AthletesNC.

## Discussion

This study is the first to investigate the effect of OC use on acute phase hormonal and systemic inflammatory parameters in elite female athletes prior to the Olympic Games. C-reactive protein concentrations were significantly elevated in AthletesOC when compared to AthletesNC. Conversely, there was no difference between the two groups for cortisol or any other marker of immune function, although there was a trend for higher IL-6 concentrations in AthletesNC.

C-reactive protein concentrations were threefold higher in AthletesOC compared to AthletesNC, which supports an earlier study (Cauci et al., 2017) that reported a fourfold increase in CRP concentrations in athletic women using an OC when compared to non-users. Interestingly, none of the athletes in the prior study (Cauci et al., 2017) recorded CRP concentrations of >10.0 mg.L^–1^ (“very high” CVD risk) (Cook et al., 2006; Cauci et al., 2017), whereas two OC users (8%) fell into this category in the current cohort. Only 16% of AthletesOC had “protective” CRP concentrations compared to 39% of AthletesNC, and 32% of AthletesOC fell into the combined “protective” and “no risk” categories (CRP < 1 mg.L^–1^) compared to 68% of AthletesNC. It should be noted, however, that CRP is only one risk factor for CVD (Ridker et al., 2005), and elite athletes would likely otherwise be considered low risk for CVD given the protective effect of regular physical activity (Li and Siegrist, 2012), low rates of excess body mass (Wd et al., 1996), and generally good nutritional practices (Lun et al., 2009; Heaney et al., 2011). Nevertheless, 36% of AthletesOC had CRP values of >3 mg.L^–1^ compared to 0% of women in the AthletesNC group, which is indicative of systemic inflammation in this cohort.

Previously, a higher percentage of athletes not using an OC were observed to have CRP concentrations of <0.5 mg.L^–1^, and fewer had concentrations of >3 mg.L^–1^, when compared to non-OC users in the general population (Cauci et al., 2008, 2017). Interestingly, this inverse relationship between CRP concentration and exercise was not observed for the athletes using an OC (Cauci et al., 2017). These findings suggest that regular exercise decreases the frequency of high-inflammatory status, while increasing the frequency of basal protective low-inflammatory status, in non-OC users but not those taking an OC (Cauci et al., 2017). Moreover, a higher percentage of AthletesOC had CRP concentrations >3 mg.L^–1^ (36%) than previously observed in OC users in the general population (31%) (Cauci et al., 2008). It is not immediately clear as to why this occurred, however, periods of intensified training can have an immunosuppressive effect (Walsh and Whitham, 2006; Gleeson, 2007). Given that blood sampling occurred 3 months prior to the Olympic Games, all athletes were performing heavy training loads which may explain the results obtained. Indeed, 87% of athletic women who were not taking an OC in the recent study by Cauci et al. (2017) reported CRP concentrations of <1 mg.L^–1^ compared to 68% of AthletesNC in the present study, indicating an increased inflammatory status even amongst non-OC users. However, in the main, the results suggest that OC users have elevated CRP concentrations relative to athletes who do not take OC, with little difference between athletes and non-athletes in terms of the CRP response to OC use.

As expected, estradiol, progesterone, and testosterone were lower in the AthletesOC group when compared to AthletesNC (Wiegratz et al., 2003; Fleischman et al., 2010). While no previous research has compared cortisol concentrations in elite female athletes according to OC status, one study found that cortisol concentrations doubled in high school/university athletes after 10 months of OC use when compared to pre-OC levels (Rickenlund et al., 2004). Although we did not have access to pre-OC cortisol data, the comparable values reported for AthletesOC and AthletesNC do not suggest a significant relationship between OC use and basal cortisol concentrations. In non-athlete populations, no consistent effect of OC use on resting cortisol concentration has been observed, with some studies showing an elevation (Timmons et al., 2005; Boisseau et al., 2013), others showing no difference (Bonen et al., 1991; Kirschbaum et al., 1996), and one showing a decreased concentration (Reinberg et al., 1996) in OC users.

No between-group difference was observed in the frequency of PBMC immune cell subsets or for any of the cytokines measured, although IL-6 concentrations were three times higher (*p* = 0.062) in AthletesNC. This was driven by significantly higher IL-6 concentrations in AthletesNC that were in the luteal phase at the time of blood sampling, when compared to those that fell into the follicular phase. Indeed, a separate analysis revealed no significant differences between AthletesNC in the follicular phase and AthletesOC. There is little consensus in the literature regarding the effect of menstrual cycle phase on IL-6 concentrations, with some studies showing elevated concentrations in the follicular phase (Angstwurm et al., 1997), others in the luteal phase (Konecna et al., 2000), and others reporting no difference (O’Brien et al., 2007). Nevertheless, these findings are interesting given that IL-6 is thought to trigger CRP expression (Black et al., 2004), yet CRP was higher in the AthletesOC group though this group displayed a trend for lower IL-6 concentrations, and there was no difference in CRP between AthletesNC in the follicular and luteal phases despite differing IL-6 concentrations. Giraldo et al. (2008) found that OC use improves inflammatory status in untrained women by lowering the concentration of IL-8 and increasing the concentration of IL-13, which was not replicated in the current findings (there was no between-group difference in IL-8 and IL-13 concentrations were below the limits of detection). No differences in IL-6 were observed between OC users and non-users in this previous study (Giraldo et al., 2008). It is important to note that almost half of the athletes in the present study had trained prior to blood collection, and thus, it is possible that the samples reflect a post-exercise cytokine response. However, separate within-group analyses of the cytokine and CRP data were performed according to training status (trained before sample collection vs. no training before sample collection), and no differences in IL-6 (or CRP) were found. This suggests that it was the use of the OC, rather than a training response, that elicited the findings observed.

Timmons et al. (2005) observed an ∼80% greater IL-6 concentration immediately post-exercise in normally-menstruating women in the follicular phase when compared to women using an OC, which indicates a blunted IL-6 response in OC users. While it is speculative to suggest an influence of OC use on the exercise-induced cytokine response in elite athletes given an exercise protocol was not employed, future studies should further examine the relationship between OC use and the cytokine response to exercise in this cohort. Although IL-6 has known inflammatory properties, increased IL-6 concentrations after exercise inhibit pro-inflammatory (i.e., TNF-α) cytokines and facilitate anti-inflammatory cytokine (i.e., IL-1ra, IL-10) production (Petersen and Pedersen, 2005), and thus, a disruption to this process may have implications for recovery and performance. A recent review by Peake et al. (2017) highlights the importance of the initial pro-inflammatory response to exercise-induced muscle injury, but notes that these interactions must be tightly regulated to avoid prolonged inflammation and tissue damage.

Animal and human studies demonstrate that regular exercise alters the balance between Th-1 and Th-2 cell subsets (Yeh et al., 2009; West et al., 2016), and initial studies indicate that OC use alters circulating white cell subsets at certain times of the menstrual cycle (Auerbach et al., 2002). Our findings indicate that, in elite athletes, OC use has little effect on key immune subsets involved in a cell mediated immune response. It should be noted that Auerbach et al. (2002) reported cell differences per liter of blood, while we reported cells relative to their parent population. However, re-calculation of our data to be comparable to the previous study (Auerbach et al., 2002) made no difference to our results (data not shown). Thus, our observation suggests OC use does not modify chronic exercise-induced PBMC immune responses in elite female athletes.

This study provides unprecedented insight into the basal immune functioning of elite athletes leading into the Olympic Games. However, given the elite status of the subjects and the pivotal timing of the study, it was not possible to manipulate the athletes’ schedules to control for factors such as training status prior to blood sampling (and the timing of blood sampling after training, for those that did train) and diet, which is a limitation of the study. Ideally, training status and/or sampling time would have been strictly controlled to ensure uniformity between groups. Nevertheless, AthletesNC and AthletesOC were very similar with respect to the number of athletes that trained/didn’t train in each group prior to blood sampling, and the timing of blood sampling post-training (2.5 vs. 2 h, respectively). Moreover, CRP and IL-6 were the only inflammatory markers assessed that were significantly different (or approaching statistical significance, in the case of IL-6) between groups, and a separate analysis of these data according to training status (trained before sample collection vs. no training before sample collection) found no differences in either IL-6 or CRP for AthletesNC or AthletesOC. This suggests that the results obtained relate to the use of OC as opposed to reflecting a post-exercise response. An additional limitation of the study was incomplete data pertaining to the OC type used by AthletesOC, thus precluding understanding of how the different OC formulas available may differentially influence inflammation. It should also be noted that the cross-sectional study design allows limited understanding of the influence exerted by OC on inflammatory markers at different time points. Nevertheless, this study highlights a substantially increased inflammatory status in athletes taking an OC at a pivotal point in their training (in the months immediately prior to the Olympic Games). As suggested by Cauci et al. (2017), it is plausible that higher basal CRP levels may result in an exacerbated inflammatory response when exposed to physical stress or injury. Future studies should investigate whether higher basal CRP concentrations are associated with reduced performance and recovery in elite female athletes.

## Conclusion

Elite female athletes selected for Olympic squads had substantially greater levels of CRP if they were users of an OC. Specifically, in the months prior to the Rio Olympic Games, AthletesOC had threefold higher CRP concentrations than their teammates who were not taking an OC. There was also a trend for lower IL-6 concentrations in AthletesOC which needs to be further explored. As female sports continue to gather momentum and public interest, future research should examine the relationship between OC use, immune function, and performance/recovery in elite female populations, as this may have important implications for the athletes’ training and recovery programs.

## Data Availability Statement

The datasets generated for this study are available on request to the corresponding author.

## Ethics Statement

The studies involving human participants were reviewed and approved by Human Research Ethics Committee – Griffith University. The patients/participants provided their written informed consent to participate in this study.

## Author Contributions

BLa contributed to the data analysis and led the manuscript writing. AC contributed to the experimental design, blood collection and handling, oversaw the blood analysis, and assisted with the preparation of the manuscript. CC contributed to the experimental design, blood handling and analysis, and assisted with manuscript preparation. MD, HM, BF, DH, NV, GW, LB, and BLu contributed to the experimental design and assisted with the preparation of the manuscript. NW contributed to the experimental design, blood collection and handling, data analysis, and assisted with the preparation of the manuscript. CM contributed to the experimental design, data analysis, and preparation of the manuscript.

## Conflict of Interest

The authors declare that the research was conducted in the absence of any commercial or financial relationships that could be construed as a potential conflict of interest.
